# Brain tumor modeling using the CRISPR/Cas9 system: state of the art and view to the future

**DOI:** 10.18632/oncotarget.8075

**Published:** 2016-03-14

**Authors:** Xiao-Yuan Mao, Jin-Xiang Dai, Hong-Hao Zhou, Zhao-Qian Liu, Wei-Lin Jin

**Affiliations:** ^1^ Department of Clinical Pharmacology, Xiangya Hospital, Central South University, Changsha, P. R. China; ^2^ Institute of Clinical Pharmacology, Central South University, Hunan Key Laboratory of Pharmacogenetics, Changsha, P. R. China; ^3^ Public Health Sciences Division/Translational Research Program, Fred Hutchinson Cancer Research Center, Seattle, Washington, USA; ^4^ Institute of Nano Biomedicine and Engineering, Department of Instrument Science and Engineering, Key Laboratory for Thin Film and Microfabrication Technology of Ministry of Education, School of Electronic Information and Electronic Engineering, Shanghai Jiao Tong University, Shanghai, P. R. China; ^5^ National Center for Translational Medicine, Shanghai Jiao Tong University, Shanghai, P. R. China

**Keywords:** brain tumors, animal models, CRISPR, oncogene, tumor suppressor

## Abstract

Although brain tumors have been known tremendously over the past decade, there are still many problems to be solved. The etiology of brain tumors is not well understood and the treatment remains modest. There is in great need to develop a suitable brain tumor models that faithfully mirror the etiology of human brain neoplasm and subsequently get more efficient therapeutic approaches for these disorders. In this review, we described the current status of animal models of brain tumors and analyzed their advantages and disadvantages. Additionally, prokaryotic clustered regularly interspaced short palindromic repeats (CRISPR)/CRISPR-associated protein 9 (Cas9), a versatile genome editing technology for investigating the functions of target genes, and its application were also introduced in our present work. We firstly proposed that brain tumor modeling could be well established via CRISPR/Cas9 techniques. And CRISPR/Cas9-mediated brain tumor modeling was likely to be more suitable for figuring out the pathogenesis of brain tumors, as CRISPR/Cas9 platform was a simple and more efficient biological toolbox for implementing mutagenesis of oncogenes or tumor suppressors that were closely linked with brain tumors.

## CURRENT STATUS: ANIMAL MODELS OF BRAIN TUMORS

Primary brain tumors have the highest mortality rates among all the cancers worldwide [1]. From the knowledge about the subtypes of gliomas, the etiology and the molecular mechanism, especially the origins [2], we learned that there were differences between rodent model of gliomas and glioblastoma in human patients. Due to the inconvenience of interference of brain tumor patients in clinical practices, it is of desperate need to develop *in vivo* animal models with brain tumors that faithfully mirror human disease, finally finding an effectively therapeutic target for treating brain tumors.

Several brain tumor models have been established as shown in Table 1. For example, it was reported that the rat glioma model could be induced by implantation of cultured glioma cells (Cell-derived xenograft: CDX) [3, 4] and transplanted tumor fragments (Patient-derived xenograft: PDX) [5]. Medulloblastom was regarded as the most common malignant brain tumor in pediatrics with a poor prognosis and *in vivo* investigation; it was often induced by transplantation of chemically modified human medulloblastoma cells such as Daoy, ONS76 and D425 [6]. Although these animal models have the advantage of making the tumor models for a short time and are widely used for the study of brain tumors, they do not comprehensively recapitulate human neoplasms and often caused the inhibitory tumor-host immunoresponses [7, 8].

**Table 1:** Animal models of brain tumors

Tumor type	Model type	Engineered drivers	Description	Ref
Glioma	CDX	HOTAIR-knock down; Bmi1- deficient	Transplantation of HOTAIR shRNA; Injection of Bmi1 -deficient astrocytes	[6-7]
Glioma	PDX	Hedgehog-responsive	Injection of Biopsy -derived glioma cells	[5]
Glioma	GEMM	Platelet-derived growth factor subunit B overexpression	Tranfection of human cells by avian sarcoma- leucosis virus	[13]
Medulloblastom	CDX	MET kinase-driven	Injection of Daoy cells to prepare intracranial xenografts	[6]

Abbreviations: CDX: Cell-derived xenograft; PDX: Patient-derived xenograft; GEMM: Genetically engineered mouse model

Besides, genetically engineered mouse models (GEMMs) are also extensively employed for investigating the pathogenesis of glioma. It was usually generated by introducing the mutations and genetic aberrations of both germ-line cells and somatic cells [9]. For instance, previous investigations illustrated that the introductions of mutant Ras protein, including HRas, VRas and NRas could trigger brain tumors [10, 11]. Postnatal PTEN loss or mutant epidermal growth factor receptor expression was also found to result in the generation of glioma in a transgenic mouse glioma model [12]. Similarly, platelet-derived growth factor subunit B overexpression contributed to the occurrence of brainstem glioma after human cells were injected into the brain stem of neonatal mouse [13]. The GEMMs has the advantage of resembling human glioblastoma as the tumor histology in the transgenic mouse is similar to that in human. However, the big disadvantage is that GEMM generation takes really long time and it is difficult to distinguish the primary mutation and the secondary mutation.

Collectively, those animal models as noted above possess respective shortages and it is essential for developing a new brain tumor model in order to more deeply probe molecular mechanisms of brain tumors and obtain better therapeutic approaches.

## CRISPR/CAS9 SYSTEM: A POWERFUL TOOL FOR GENOME EDITING

The recently mentioned prokaryotic clustered regularly interspaced short palindromic repeats (CRISPR)/CRISPR-associated protein 9 (Cas9) system is a powerful genetic engineering tool in which guide RNA (gRNA) targets the programmable nuclease Cas9 to a desired genomic DNA sequence and Cas9 precisely cleaves both strands of interest [14-18].

**Figure 1:**
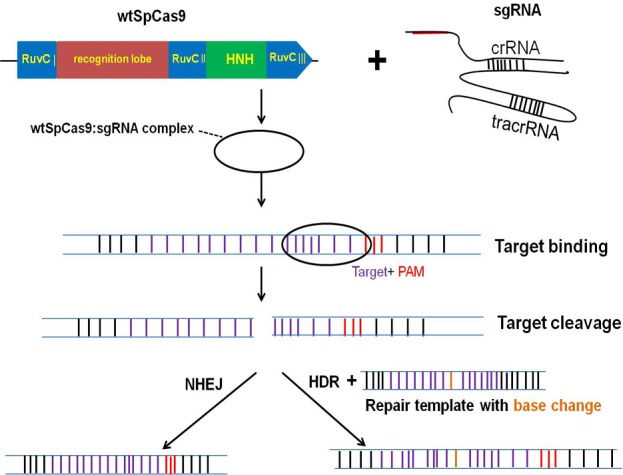
The CRISPR/Cas9 system for genome engineering. The CRISPR is composed of two major components including a CRISPR-associated endonuclease (Cas9) and a single guide RNA (sgRNA) The Cas9 from S. pyogenes (wt SpCas9) is shown in this figure as it is the most widely used in genome editing nowadays. After wt SpCas9 and sgRNA form a riboprotein complex, they can bind any genomic sequence with a protospacer adjacent motif (PAM), directing DNA double-strand breaks (DSBs) at the target site. DSBs are then repaired by either non-homologous end-joining (NHEJ) or homology-directed repair (HDR) pathway.

**Figure 2:**
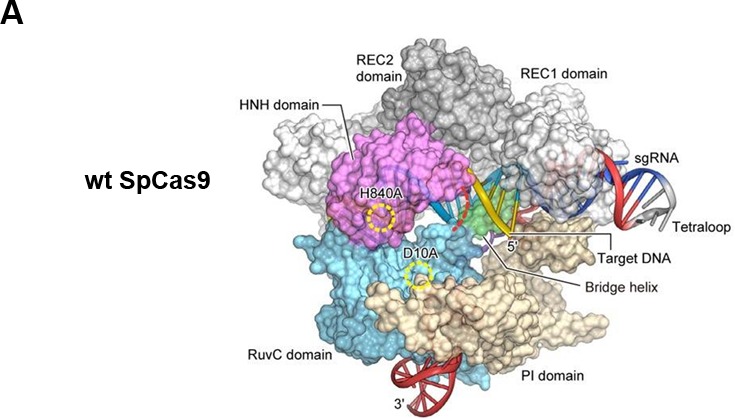

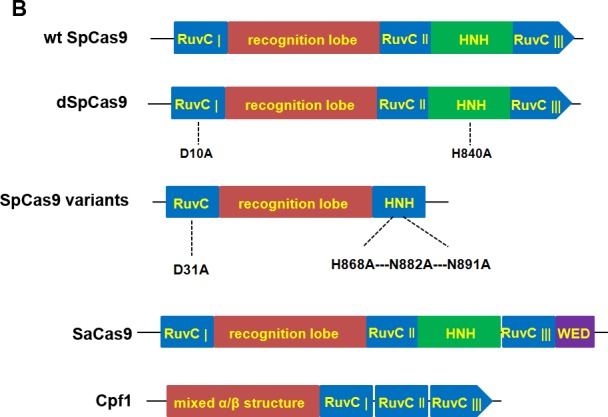

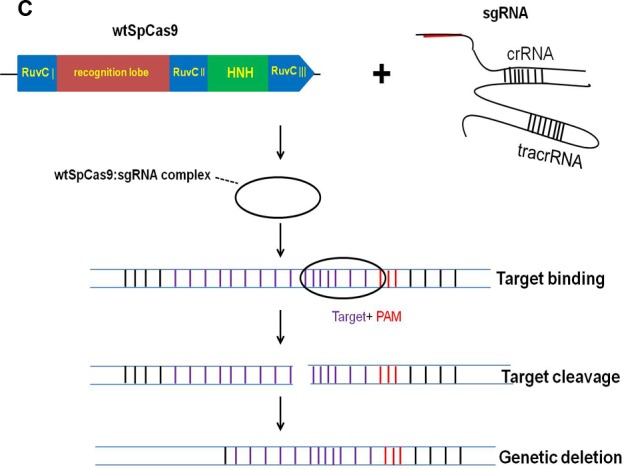

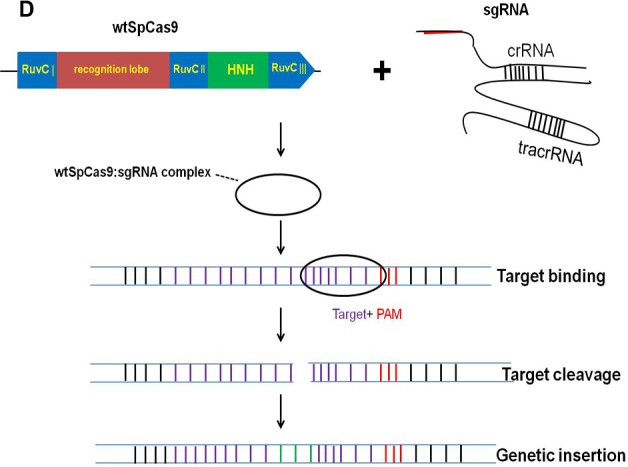

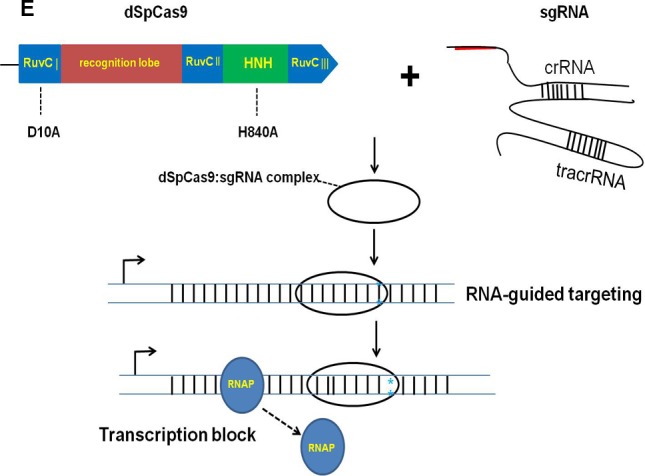

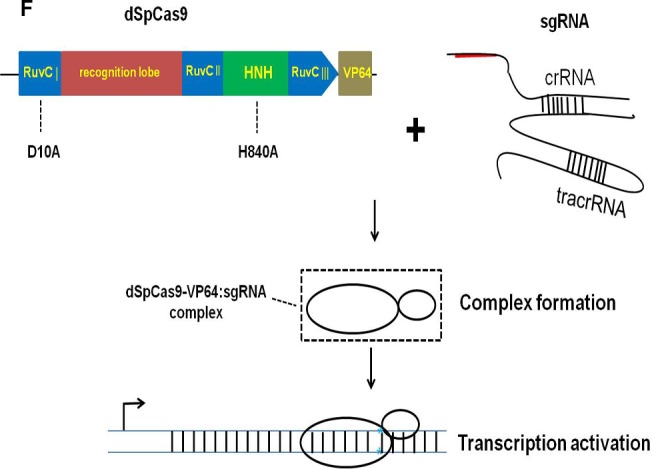

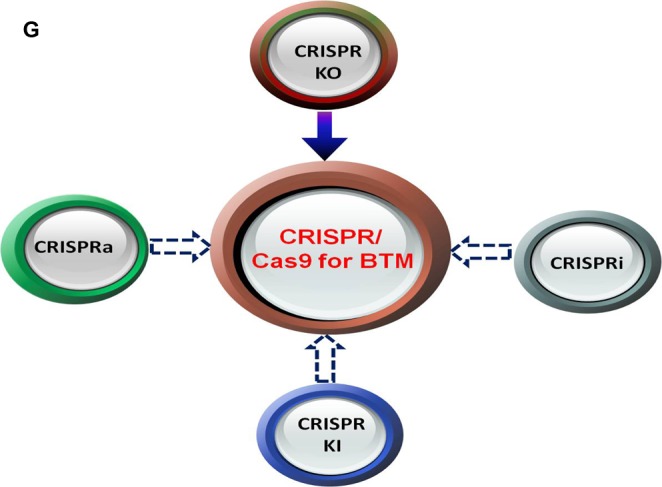
Proposed applications of the CRISPR/Cas9 system for brain tumor modeling **A.** shows the crystal structure of wt SpCas9 referred to Zhang et al [70]; **B.** is the model graphs of wt SpCas9, dSpCas9, SpCas9 variants, SaCas9 and Cpf1; **C. D. E. F.** display the processions of four different types of CRISPR-mediated genome editing including CRISPR knock out (CRISPR KO), CRISPR knock in (CRISPR KI), CRISPR interference (CRISPRi) and CRISPR activation (CRISPRa), respectively; **G.** proposes four different sorts of CRISPR/Cas9 techniques are possibly involved in brain tumor modeling (BTM). CRISPR KO is presently successfully applied for BTM (as shown by solid arrow). We propose CRISPR KI, CRISPRi and CRISPRa are used for BTM (as shown by dotted arrows).

As shown in Figure 1, the genomic editing for CRISPR/Cas9 system mainly requires two biological components: Cas9 and an engineered single guide RNA (sgRNA). The sgRNA is composed of both a CRISPR RNA (crRNA) component and a trans-activating crRNA (tracrRNA). SgRNA recognizes the complementary genomic DNA sequences flanked by a protospacer adjacent motif (PAM), which is made up of NGG or NAG trinucleotide for Cas9 [19]. After Cas9 is combined with a sgRNA which is complementary to a target DNA sequence, the double-stranded break (DSB) is formed and then repaired by either non-homologous end-joining (NHEJ) or homology-directed repair (HDR) pathway [20]. Figure 2A and Figure 2B showed the crystal structure and model graph of wild type Cas9 from S. pyogenes (wt SpCas9). Additionally, the catalytically dead Cas9 mutant (dSpCas9) and other SpCas9 variants were also indicated in this Figure 2B. These SpCas9 mutants were shown to significantly minimal off-target effects [21, 22]. A small Cas9 from Staphylococcus aureus (SaCas9) was also recently obtained for eukaryotic genome engineering [23]. SaCas9 shared only 17% of identical sequence with SpCas9 [23]. And smaller size of SaCas9 makes it easier deliver to somatic tissues for genome editing, compared with SpCas9 (Figure 2B). The centromere and promoter factor 1 (Cpf1) was hypothesized to be the effector of a CRISPR locus that was different from the Cas9-containing class 2 CRISPR system [24]. Structurally, Cpf1 is lack of a second HNH endonuclease domain, which is inserted within the RuvC-like domain of Cas9, as shown in Figure 2B. It was previously reported that Cpf1was a CRISPR-associated two-component RNA programmable DNA nuclease and exhibited robust nuclease activity in human cells [24].

RNA-guided nucleases Cas9 employs simpler, Watson-Crick base-pairing rules between the sgRNA and the genomic DNA sequence of interest, compared with the early method including zinc finger nucleases (ZFNs) and transcription activator-like effector nucleases (TALENs), which acquire the targeting genomes *via* protein-DNA interactions [25-27]. Although ZFNs and TALENs are very beneficial for performing precise genome editing, their applications have been limited as a result of high cost and difficult design of this endonucleases. The RNA-guided endonucleases Cas9 from the microbial adaptive immune system CRISPR can be easily targeted to any selected genomic location by a short RNA guide.

## PROPOSED APPLICATIONS OF THE CRISPR/CAS9 SYSTEMS IN BRAIN TUMOR MODELING

Modeling cancer including brain tumors in mice through genetic manipulation in the germline of an organism has long been regarded as the gold criteria for seeking putative oncogenes or tumor suppressor genes (TSGs). Loss-of-function mutations in TSGs and gain-of-function mutations in a proto-oncogene were reported to be involved in the generation and progression of glioma [28, 29]. Traditional cell-type-specific knockout techniques *via* homologous recombination are shown to cause the loss of function for TSGs in embryonic stem cells, finally leading to the malignant transformation [30]. Nevertheless, the low efficiency of homologous recombination and the time-consuming property for the generation of GEMMs hamper its applications. As alternatively, the CRISPR/Cas9-guided endonuclease technique provides more efficient and precise modification of the DSB sites at target genomes [31]. One big advantage of CRISPR/Cas9 is that it takes short time to generate GEMM model. Nowadays, this versatile genome editing technique has been developed for producing gene knockout models of various animals including mouse [32], rat [33-35], as it can more precisely understanding human diseases than the conventional gene knockout models.

In general, CRISPR/Cas9 system contains four types of genome editing techniques including CRISPR knock out (CRISPR KO), CRISPR knock in (CRISPR KI), CRISPR interference (CRISPRi) and CRISPR activation (CRISPRa) (Figure 2C-2F). Using the CRISPR KO-mediated target PTEN efficiently diminished PTEN expression in neurons, inducing neuronal hypertrophy and altering neuronal excitation, while targeting NF1 was shown to facilitate astrocytogenesis and combined targeting of three TSGs (including PTEN, NF1 and P53) by multiplex CRISPR/Cas9 triggered the pathogenesis of glioblastoma [36]. Besides, CRISPR KO technology was also previously found to efficiently and completely delete two well-known synaptic proteins including GluN1 subunit of the NMDA receptor and the GluA2 subunit of the AMPA receptor in neurons [37].

Additionally, knockin animal models has been successfully prepared using CRISPR/Cas9 system (which referred to CRISPR KI) [38-42]. It was previously found that Cre-dependent Cas9 konckin mice exhibited indel formation near predicted cleavage site on *NeuN* (a neuron-specific marker) locus indels in the brain *via* virus-mediated sgRNA expression as reported by J. Platt [38]. Delivery of a single adeno-associated virus (AAV) vector in the lung caused the genetic mutations of p53, LKB1 and KRAS using the Cas9 knockin mouse, resulting in the macroscopic tumors of adeno-carcinoma pathology [38]. The feasibility of direct mutations of TSGs and oncogenes was also reported in the liver by the CRISPR/Cas system [43]. What's more important, recent investigations illustrated that the somatic gene transfer of CRISPR plasmids encoding Cas9 and gRNAs was suitable to induce distinct brain tumors including sonic hedgehog medulloblastoma and glioblastoma [44].

CRISPR/Cas9-mediated control of gene suppression (which referred to CRISPRi) is also an important application for this genetic engineering technology. The CRISPRi system, derived from the Streptococcus pyogenes CRISPR pathway, is established by the coexpression of catalytically incactive Cas9 (dCas9) fusion proteins and a customizable sgRNA [15, 45-47]. As dCas9 is lack of endonuclease activity and generates a targeted protein-RNA complex when coexpressed with a guide RNA. This Cas9-sgRNA complex binds to DNA sequences complementary to the sgRNA and specifically blocks transcription elongation within protein-coding regions, RNA polymerase binding, or transcription factor binding. It was previously found that usage of CRISPRi could remarkably suppress expressions of any target gene of interest in Escherichia coli, with no obvious off-target effects [46]. An additional investigation revealed more efficient gene suppression in eukaryotes by dCas9 fused with a transcription repression domain or exogenous transgene activation [45]. The multiple target genes were also evidently repressed in human cells *via* this system. In fact, other targeted gene modulation methods have been widely established in the past few years including RNA interference (RNAi) [48] and polydactyl zinc-finger proteins [49-51] and sequence-specific transcription activator-like effector (TALE) [52-54]. Among these traditional methods, RNAi is a classical technology for perturbing target genes on the mRNA level by designing complementary RNAs [28], but it is limited by evident off-target effects, low efficiency, serious toxicity and small-scale use in particular organisms [55]. The CRISPRi technology is more efficient and has the minimal off-target effects due to its simple and precise design. Engineered DNA-binding proteins such as custom zinc-finger or TALE proteins provide a very useful platform for achieving diverse targeted regulatory functions when combined with effector domains. Nevertheless, this method is a time-consuming process and the construct development requires high cost. As a result, it is likely to be very difficult to build a comprehensive protein library to simultaneously perturb multiple target genes [56]. In contrast, the CRISPRi method is very convenient and cheap for suppressing target genes due to use of sgRNA with a specific 20-nt-long complementary region. And with the faster and cheaper synthesis of wide-range DNA oligonucleotide, it allows for targeting large amounts of genes to probe gene function using CRISPRi system.

Furthermore, dCas9 could also be fused to transcription activation domains to form RNA-guided transcriptional activator system (which referred as CRISPRa), finally regulating gene expression *via* targeting the promoter region of endogenous genes [57, 58]. Usage of dCas9-based transcription activators was observed to result in the up-regulation of several endogenous loci [25]. In human cells, it was also illustrated that the dCas9-VP64 fusion protein was found to be directed by single or multiple gRNAs and produce robust transcriptional activations of endogenous human genes such as VEGFA [58]. This CRISPRa system was also reported to powerfully up-regulate multiple exogenous reporter genes in both human and mouse transformed cells as well as in embryonic stem cell cells in a tunable manner [59]. Endogenous IL1RN, SOX2, and OCT4 genes were also observed to be activated in human embryonic kidney 293T (HEK293T) cells *via* CRISPRa technology [59]. It implicates that CRISPRa is likely to be used to target heterologous effector domains in human cells. In a word, CRISPR KO and CRISPR KI are more effective for making genetic mutations while CRISPRi and CRISPRa for genetic expressions. Besides, the latter two techniques seem to interference lncRNA more efficiently.

According to these findings as mentioned, we propose different types of brain tumors modeling can be established using CRISPR/Cas9, as displayed in Figure 2G. CRISPR/Cas9-mediated brain tumor modeling is likely to be more suitable for exploring the pathogenesis of brain tumors, as CRISPR/Cas9 platform is a simple and more efficient biological toolbox for implementing mutagenesis of oncogenes or tumor suppressors that are closely linked with brain tumors. Indeed, prior investigations supported the notion that the preparation of brain tumor modeling could be carried out *via* CRISPR knock out (CRISPR KO) technology [36, 44, 60-62], which were partly in line with our hypothesis that CRISPR technology was served to establish efficient knock-out brain tumor models. Recently, Zhang et al also emphasized that CRISPR/Cas9-mediated precise and efficient genome editing could help to deeply figure out the logic of neural circuits and disclose the mysteries of diverse neurological diseases including brain tumors [63].

## FURTHER REQUIRED CLARIFICATION FOR CRISPR/CAS9 TECHNIQUE

Despite great progress has been made in the past two years with the CRISPR/Cas9 system and the establishment of diverse cancer models, there are still some issues that require further clarification.

Firstly, the delivery of Cas9 for establishing somatic mutation of mouse models should be further improved. It was previously demonstrated that efficient delivery of genome-editing proteins could significantly enhance Cas9-mediated genetic engineering of target sequences [64, 65]. Split Cas9 was also reported to be beneficial for *in vivo* genome editing in HEK293FT cells [66, 67]. Finding smaller Cas9 orthologs could also improve CRISPR delivery as a result of facilitating viral vector package [23, 68, 69]. However, it is still essential to optimize the methods for efficient delivery and expression of CRISPR-Cas9 system in order to be suitable for each cell-type or organism. Since some cell types or tissues are resistant to transfection or infection by viral vectors, researchers should develop methods that alter the expressions of Cas9 endonuclease or gRNAs that is specific to a tissue or cell type.

Secondly, the specificity of genetic modification is also an important issue to be considered. As targeting by CRISPR/Cas9 is dependent on nearly 23 base pair matches [70], it may generate many nonspecific mutations in the genome editing. Great efforts should be made to improve the ratio of on- and off-target effects. Several research groups have developed new technologies to maximally diminished off-target genome editing such as truncated sgRNA [71, 72] and dCas9-Fok I fusions [73]. Additionally, employment of bioinformatic screening and paired Cas9 nickases were also found to remarkably enhance genome editing specificity [74, 75]. Cas9 off-target sites have been reported by Genome-wide analysis [76-78]. Evaluation of off-target effects is a critical step of developing this method. Although reduced off-target effects are observed by tru-gRNAs and paired nickases, further improvements will be required, especially for therapeutic interferences.

Another issue to be concerned is the safety of the CRISPR/Cas9 platform [79]. As this genome engineering technology has the unparalleled potential for modifying human and nonhuman genomes, it is likely to confound unknown risks to human health. Research is required to understand and avoid risks when using the CRISPR/Cas9 technique.

## CONCLUSION REMARKS AND FUTURE PERSPECTIVES

Although brain tumors have been known for more than 50 years, there are still many problems to be solved. The etiology of brain tumors is elusive and the treatment is not satisfactory [80]. There is in great need to develop suitable brain tumor models that faithfully reflect the etiology of human brain neoplasms.

Traditional animal models of brain tumor possess various faults. For example, the cells transplanted in the CDX model are mainly high-grade origin and this model can not comprehensively mimic different grades of brain tumors. In terms of the GEMM model, the generation is a low-efficient and time-consuming process. The CRISPR/Cas9 system can be served as a novel tool to overcome the shortages of the existing methods. It can precisely and efficiently obtain the genetic mutations of the target genome at a selected DNA site. In central nervous system, previous investigations illustrated that animal models of sonic hedgehog medulloblastoma and glioblastoma have been successfully established using CRISPR KO [81, 82]. Table 2 listed several regulatory factors (*p53*, *Nf1*, *Pten*, *Ptch1*, *Bmi1*, *Met*, *Notch1*, *CDK6*, miR-10b, TERT, LSD1, miR-218 and miR-128) which were closely related to the etiology of brain tumors [28, 43, 44, 83-92]. It was found that Cas9-deletion of multiple TSGs including *p53*, *Nf1*, *Pten* and *Ptch1* could induce the formation of brain tumors [44]. We propose that other regulators that are associated with the generation of brain tumors can be manipulated by CRISPR/Cas9 system.

**Table 2:** Representative factors that can be manipulated by different types of CRISPR genome editing technologies for brain tumor modeling

Factors	Role in tumorigenesis	Proposed CRISPR methods	Previous methods	Ref
p53 ^(a) (b)^	tumor suppression	CRISPR KO	CRISPR KO	[43, 44]
Nf1 ^(a)^	tumor suppression	CRISPR KO	CRISPR KO	[44]
Pten ^(a) (b)^	tumor suppression	CRISPR KO	CRISPR KO	[43, 44]
Ptch1 ^(c)^	tumor suppression	CRISPR KO	CRISPR KO	[44]
Bmi1 ^(a) (d)^	tumor facilitation	CRISPR KI	Bmi1 shRNA	[83, 84]
Met ^(e)^	tumor facilitation	CRISPR KI	CRISPR KO	[85]
Notch1^(a)^	tumor facilitation	CRISPR KI	Notch1 siRNA	[86]
CDK6^(a)^	tumor facilitation	CRISPR KI	CDK6 shRNA	[87]
miR-10b^(a)^	tumor facilitation	CRISPRa	miR-10b mimics	[88]
TERT^(e)^	tumor facilitation	CRISPRa	CRISPRa	[90]
LSD1^(f)^	tumor facilitation	CRISPRa	CRISPRa	[90]
miR-218^(a)^	tumor suppression	CRISPRi	anti- miR-218	[28]
miR-128^(a)^	tumor suppression	CRISPRi	anti-miR-128	[91]

Abbreviations: CRISPR KO: CRISPR knock out; CRISPR KI: CRISPR knock in; CRISPRa: CRISPR activation; CRISPRi: CRISPR interference.

Note: (a) glioma; (b) liver; (c) medulloblastoma; (d) breast cancer with brain metastases; (e) HEK293 cell line; (f) embryonic stem cell

Furthermore, the CRISPR/Cas9 technology has been widely used for genome engineering in many stem cell organoids including stomach [93, 94], intestine [95-97] and pancreas [38, 98, 99]. In details, the locus of the cystic fibrosis transmembrane conductor receptor in cultured intestinal stem cells derived from cystic fibrosis patients was accurately repaired using CRISPR/Cas9 genome editing system *via* homologous recombination [97]. Efficient gene transfer was also successfully established in human intestinal organoids by this platform [96]. Human pluripotent stem cells (hPSCs) such as induced pluripotent stem cells (iPSCs) and embryonic stem cells (ESCs) are considered as very useful tools for elucidating regulatory processes during early development and the pathogenesis of genetic disorders [100, 101]. Since the human iPSCs retain the individual genetic information, combination with this cell and CRISPR-mediated genetic editing may be critical for exploring this phenotype that appears during cell differentiation [63]. Although traditional knockout of genes related to hPSCs self-renewal or survival may block cell propagation and survival, a recent investigation reported that successful knockout of multiple genes including SOX2, PAX6, OTX2 and AGO2 was established in human iPSCs and ESCs using CRISPR system [102]. In the future, CRISPR/Cas9 system is likely to apply for studying human brain tumors in human iPSCs derived from brain tumor patients. The large genomic mutation databases generated from sequencing of the GBM patient indicate a lot new gene mutations can potentially response for glioma formation. With the CRISPR/Cas9 tool, we can also easily generate a new model based on the information we learned from this database in a more effective way.

Although the development of CRISPR/Cas9 technology for genome editing raises some social challenges to some extent and possibly brings out a series of uncertainty and fear of catastrophic misuse, it can never cease to inspire us for investigating the precise molecular mechanisms of brain tumors using this biological tool.

## Abbreviations

CDX: cell-derived xenograft
PDX: patient-derived xenograft
GEMM: genetically engineered mouse model
CRISPR: clustered regularly interspaced short palindromic repeat
Cas9: CRISPR-associated protein 9
gRNA: guide RNA
sgRNA: single guide RNA
crRNA: CRISPR RNA
tracrRNA: trans-activating crRNA
PAM: protospacer adjacent motif
DSB: double-stranded break
NHEJ: non-homologous end-joining
HDR: homology-directed repair
dSpCas9: dead Cas9 mutant
SaCas9: Staphylococcus aureus Cas9
Cpf1: centromere and promoter factor 1
ZFN: zinc finger nuclease
TALEN: transcription activator-like effector nuclease
TSG: tumor suppressor gene
CRISPR KO: CRISPR knock out
CRISPR KI: CRISPR knock in
CRISPRi: CRISPR interference
CRISPRa: CRISPR activation
RNAi: RNA interference
TALE: transcription activator-like effector
hPSC: human pluripotent stem cells
iPSC: induced pluripotent stem cell
ESC: embryonic stem cell
